# Impact of Universal Vaccination Against Hepatitis B: The Italian Model

**DOI:** 10.5812/hepatmon.6396

**Published:** 2012-07-30

**Authors:** Rosa Cristina Coppola, Angelo Meloni, Marcello Campagna

**Affiliations:** 1 Department of Public Health, Clinical and Molecular Medicine, University of Cagliari, Monserrato, Italy

**Keywords:** Vaccination, Hepatitis B Virus, Italy

In the 70s and 80s, Italy was one of the countries with the highest prevalence of hepatitis B in Europe; the overall endemic of hepatitis B virus (HBV) was classified as intermediate, yet it resulted from a dual pattern of high endemic in south Italy and of low endemic in most of north Italy parts. The mean countrywide rate of carriers of the HB surface antigen (HBsAg) was 2.5 %; however figures were as high as 10 % in areas of south Italy. HBsAg rate in pregnant women was 2.4 % in average; however in some areas as many as 5 % of the women were carriers [1]. Screening of pregnant women during the last three months of pregnancy was recommended from the early 80s; only one third of women were initially screened, but the percentage increased at the end of the decade. The incidence of hepatitis B in the mid of the 80s was over 40 cases per 100.000 young italian (15-24 years old), around 10 cases/100.000 among subjects who aged over 24 years and 5 cases per 100.000 in children aged up to 14 years. Starting from the mid-1980s, the incidence of hepatitis B progressively declined, as a return of the alert to the infection with the human immunodeficiency virus (HIV), the changing of social behavior, the introduction of public health measures such as refinement in blood screening, the use of universal precautions in medical settings and the implementation of vaccination [1][2][3][4][5][6]. The first step in achieving active HBV prophylaxis in the country was the recommendation in 1983 to selective immunizes groups at risk for HBV infection. In 1991 hepatitis B vaccination became universal with a program based on a two cohort’s strategy: vaccination became mandatory for all newborns and for all 12 years old adolescents. It was programmed for 12 years until 2003 when the two cohorts joined. In parallel, screening of pregnant women in the third trimester of pregnancy was also made mandatory and vaccination continued to be offered free of charge to risk groups [7]. In 2003 when the two cohorts of the vaccination program bonded together all the population from zero to 24 years was immunized by vaccination. At this time the Italian Health Authority decided to stop vaccination in the 12 years cohort and to continue in newborns. Since then and up to now, 179 countries have enforced hepatitis B birth vaccination [3][8][9][10]. New questions have been raised in the meanwhile: how long anti-HBs remain detectable, how long vaccine-induced immunity is expected to last, is the finding of anti-HBs an obligatory expression of immunity to HBV. Answering these questions a multicenter study was carried out in 2003, involving more than 1200 children who were vaccinated at birth and were enrolled into the study 11 years later. They were tested for HBV markers (HBsAg, anti-HBs and anti-HBc) and none turned out positive to HBsAg. Only one subject was positive to anti-HBc: he was negative to HBV-DNA and had an anti-HBs titer higher than 1000 mIU/ml. Overall 64 % of the vaccines had anti-HBs titers higher and 27 % lower than 10 mIU/ml respectively and 9 % were negative. Second part of the study was controling of 590 recruits who received a primary course of vaccination in 1991 when they were 12 years old. The majority (87 %) had high antibody titers; 4 had anti-HBc without sign of active infection, 8.5 % had titers lower than 10 mIU/ml and 4 % were negative [11]. The data from Italy corresponded to the worldwide experience; in 15-50 % of children responded to a primary course of vaccination the titer of anti-HBs diminished or the antibody became undetectable after five to 15 years [12].

The long term vaccine immunity can be measured by determining the anamnestic response of anti-HBs after challenging with a booster dose of vaccine. Among the cohort vaccinated at birth and tested after 11 years from the primary course, an anamnestic response (anti-HBs > 100 mIU/ml) after the booster dose occurred in 86.3 % of the vaccines, particularly in 65 % and 94.5 % of the those negative and with a baseline anti-HBs titer < 10 mIU/ml respectively. Likewise, 83.6 % of recruits who received a booster dose exhibited an anamnestic response and overall only 2.1 % of recruits (11/520) were recommended to receive a new full course of vaccination. All those receiving a new full course of vaccination developed an anti-HBs response [12]. Thus, the evidence from the Italian study in analogy with evidence from the scientific literature worldwide [3][5][12] confirmed that in adolescents who received vaccination at birth, immunity is maintained until the time when risk behavior may be expected; the longest available follow up study was carried out in Taiwan through 23 years, with over 75 % of vaccines showing an anamnestic response [13]. The conclusion is that there is no need for booster vaccination to sustain immunity; it seems therefore more appropriate to locate economic resource in increasing new vaccinations than in booster doses [14]. Another parameter commonly used to assess the long- term protection of HBV vaccine is the rate of breakthrough infections. Breakthrough HBV infection is defined by the development of anti-HBc, without chronic infection. Infection with acquisition of HBsAg has been reported in one health care worker and in infants of carrier mothers, correlated with a high maternal viral load and/or HBeAg presence, as well as. Recently 15 years follow-up studies showed that the prevalence of breakthrough infections has ranged from 0 % to 17.7 % in the general population and that the prevalence in children of carrier mothers was up to 33.3 % [15][16]. Overall, few clinically significant infections and few new carriers were detected [17][18][19]. A 24 years study in Gambia reported a 67 % vaccine efficacy against the raising of anti-HBc and of 97 % against the development of the HBsAg carriage status; the risk of hepatocellular carcinoma attributable to HBV at age 50 was 70 % – 80 % lower than in historical cohorts [20]. However, the definition of breakthrough infection must be distinguished whether it means the failure of post-exposure prophylaxis, the occurrence of HBV infections in vaccinated subjects who responded to vaccine, the development of HBV infections in non-responders or evidence of the transient presence of HBsAg, HBV DNA and sero conversions to anti-HBc. Overt HBV infections in fully vaccinated subjects occur rarely and much rarely result in acute self-limiting hepatitis. Only five-six cases of acute disease are notified per year in Italy in the population of 16-17 million which have undergone vaccination over the last 20 years [2]. The virological features often are not known but the genotype of HBV or genetic factors of the host may play a role [14]. Lymphocytes T and B cell activity is also relevant in understanding the long term protection after HBV vaccination. The memory of the B lymphocytes, elicited through vaccination and maintained over time, is involved in long-term immunity and protection against HBV, independence of the serum levels anti-HBs; the memory of T and B cells for the HBsAg is present in all vaccines even in the absence of detectable anti-HBsAg (ELISPOT after 10 days) [21][22].

The long-term protection is commonly measured with four methods. Sero-epidemiological and LT vaccine studies disclosed different carrier rates in Taiwanese university students of 9.8 % before vaccination and 0.8 % in vaccinated cohort in a 23 years follow up [23][24]. Other studies in the same region showed an average annual incidence of hepatocellular carcinoma in 0.7 cases/100,000 six to14 years children in 1981–1986 and 0.36 cases/100,000 in 1990–1994 [25] and a 20 years follow up study in the same region found no new carrier in the different vaccinated birth cohorts [26]. In Italy the coverage rate of HBV vaccination (third dose) in children younger than 24 months of life (2000-2007) increased from 94.1 % in 2000 to 96.4 % in 20076 while the Incidence of acute HBV hepatitis decreased markedly from 1985 to 2010 in the age classes 0-14, 15-24 and > 25 years; this trend was also recently confirmed [27]. The remaining question in the current scenario is whether booster vaccination is needed to sustain immunity. Available data suggest that HBV Vaccine provides an enduring protection due to the strong immunological memory that persists more than 10 years after immunization of infants and adolescents with a primary course of vaccination. Therefore booster doses of vaccine are not necessary to ensure long-term protection [3][11]. This was recently confirmed by Leuridan and Van Damme who stated that there is no need for boosters in immunologically competent persons as long as a full vaccination course was adequately administered. A booster dose should be eventually planned for immune compromised patients, based on serological monitoring [14]. Decisions on an additional hepatitis B vaccine 10, 20 or 30 years later should be based, not on the amount of remaining anti-HBs, but on the appearance of disease in the population. Boosting should not be considered until we understand the meaning of the non-detectable antibodies, especially as antibodies decline rapidly after boosting. Monitoring coverage is not sufficient and serological surveys of HBV infection markers (as primary end-points) are needed, supplemented by acute disease surveillance and follow up of long-term cohorts of vaccines. Future holding for the Italian vaccination policy are the maintenance of mandatory vaccination of infants and HBsAg tested pregnant women, the increase of vaccination coverage in high risk groups (households contacts of HBsAg carriers, health care workers), and the active offer for vaccination in women and offspring of immigrated populations.
